# Pain Catastrophizing Is Related to Static Postural Control Impairment in Patients with Nonspecific Chronic Low Back Pain: A Cross-Sectional Study

**DOI:** 10.1155/2020/9629526

**Published:** 2020-10-28

**Authors:** Chanjuan Zhang, Zhou Zhang, Yuelong Li, Chenyang Feng, Haiqi Meng, Yang Gao, Wai Leung Ambrose Lo, Chuhuai Wang

**Affiliations:** ^1^Department of Rehabilitation Medicine, The First Affiliated Hospital, Sun Yat-sen University, Guangzhou 510080, China; ^2^Department of Information, Sun Yat-sen University Cancer Center, Guangzhou 510060, China; ^3^Guangdong Engineering and Technology Research Center for Rehabilitation Medicine and Translation, The First Affiliated Hospital, Sun Yat-sen University, Guangzhou 510080, China

## Abstract

**Purpose:**

Pain catastrophizing may contribute to the altered trunk muscle activity in patients with nonspecific chronic low back pain (NSCLBP). It is unclear if pain catastrophizing influences static postural control in patients with NSCLBP. This study aimed to investigate the relationship between pain catastrophizing and static postural control in NSCLBP patients.

**Methods:**

Sixty-eight participants with NSCLBP and 40 healthy participants were recruited. Postural control was assessed by the sway area and the sway length of the center of pressure (COP) during balance tests. Pain catastrophizing in participants with NSCLBP was assessed by the Pain Catastrophizing Scale (PCS). Bilateral transversus abdominis (TrA) activation was evaluated by ultrasound imaging-measured percent change in muscle thickness. Associations between COP parameter and PCS/subscales of PCS were examined by multiple linear regression (MLR).

**Results:**

Our results observed a larger COP sway area in NSCLBP group under eyes-closed condition (*p* < 0.001) and a lower level of voluntary activation of the bilateral TrA (*p* < 0.001), compared with the healthy control group. The MLR analyses revealed that the COP area sway under eyes-closed condition was significantly associated with the PCS score/helplessness score of PCS, voluntary activation of the left TrA, and age in participants with NSCLBP (*β* = 0.222/0.236, 0.341/0.344, and 0.328/0.325; *p*=0.045/0.033, 0.002, and 0.004, resp.).

**Conclusions:**

Static postural control was associated with pain catastrophizing, voluntary activation of TrA, and age in participants with NSCLBP. This indicated that pain catastrophizing may affect postural control and should be considered when interpreting balance test results and managing NSCLBP.

## 1. Introduction

Chronic low back pain (LBP) is considered as the leading cause of disability. It has a high lifetime prevalence worldwide, affects people of all ages, and causes tremendous economic and societal burden [1, 2]. Up to 90% of chronic LBP is classified as nonspecific LBP (NSCLBP) with no known pathoanatomical cause [2]. NSCLBP is multidimensional in nature, with various factors contributing to its onset and persistence [2]. The negative cognitive-emotional factors predispose the person into the development of LBP [3]. The strengthened corticostriatal functional connectivity may contribute to the transition of acute LBP to chronic LBP [4], and the motor control alteration may play a role in the persistence or chronification of LBP [2]. Among these factors, trunk postural control impairment has been suggested to be a contributing factor to NSCLBP [2, 5].

Postural control is responsible for spine stability, posture, and movement. It is fundamental for bearing loads and avoiding injury and pain [2]. Body sway, as measured by center of pressure (COP) trajectories, is widely used to assess postural control [6]. COP is an index of postural stability during standing: larger COP sway indicates greater postural instability [6, 7]. Existing studies have indicated a general tendency for increased COP sway in static balance tests among patients with LBP when compared with healthy controls; however, it is not always increased, as some studies have found no differences or reduced COP sway in patients with LBP [6, 8]. According to the spinal stability model, postural control of the trunk is dependent on a constant interplay between sensorimotor information and motor outputs to the active zone (muscles) and the body's level of control over the passive zone (osteoligamentous spinal structures) [2, 9]. The altered recruitment or activation of the active zone (muscles), such as the transversus abdominis (TrA), in patients with spinal pain is commonly estimated by the ultrasound image-measured percent change in muscle thickness [10–12]. An ultrasound imaging study indicated a moderate correlation between postural balance and the thickness of the TrA during rest and abdominal drawing-in manoeuvre (ADIM) in participants with chronic LBP [10]. The central nervous system receives reduced proprioceptive feedback from the spinal tissues as results of the altered muscle recruitments. This in turn induces inaccurate estimations of the center of mass. Inappropriate muscle responses and altered postural control mechanisms are subsequently generated which contribute to postural instability [2, 13].

In the catastrophizing-based fear-avoidance model, because of the overestimation of the threat value or seriousness of painful stimuli, people with greater pain catastrophizing are more likely to tighten the spine to avoid excessive spinal movement and reduce the risk of injuries [5, 14, 15]. Pain catastrophizing is characterized by the tendency to exaggerate the threat value of a pain stimuli, the interpretation of pain as insurmountable, and helplessness in the context of pain or the anticipation of pain [14]. Defined as persistent negative cognitive and emotional responses to actual or anticipated pain, pain catastrophizing consists of rumination, magnification, and helplessness [14]. Pain catastrophizing is the precursor and cognitive element of pain-related fear and refers to the process during which pain is conceived as being extremely threatening [16]. As a related mechanistic contributor to the experience of LBP, increased pain catastrophizing has been demonstrated to be associated with increased pain sensitization and decreased sensitivity to innocuous stimuli in individuals with chronic LBP [17]. Mazaheri et al. [18] found no or a minimal effect of fear of pain (assessed by the Pain Catastrophizing Scale (PCS) and Tampa Scale for Kinesiophobia) on postural control in patients with subacute LBP (duration ≥ 6 weeks). However, chronic LBP differs from subacute LBP. Compared with subacute LBP, chronic LBP is more likely to exert secondary effects on cognitive–emotional interactions including anticipating the consequences of persistent pain to the future well-being and life [19, 20], which may be specifically manifested in pain catastrophizing [21]. The conclusion deduced from those studies on subacute LBP may be inapplicable to the chronic LBP population. It was proposed that the postural control adoption varied among individuals, and the cognitive-emotional factors might alter postural control in people with chronic LBP [2, 5]. To date, there is a lack of empirical evidence that demonstrate the impact of pain catastrophizing on postural control in people with NSCLBP. It remains unclear if pain catastrophizing may influence postural control in people with NSCLBP.

This aims of this study were to (a) estimate whether static postural control was impaired in patients with NSCLBP by comparing the COP parameters between NSCLBP patients and healthy controls and (b) investigate the relationships between pain catastrophizing and static postural control in people with NSCLBP.

## 2. Materials and Methods

### 2.1. Participants

The inclusion criteria for the NSCLBP group were as follows: (1) has been clinically diagnosed with NSCLBP with intermittent or persistent pain from T12 to the buttocks that has lasted for at least 3 months [1, 15], (2) is between 18 and 65 years old, (3) reported to have a minimum score of 2 on the Visual Analogue Scale (VAS) [22], (4) right-hand dominant, and (5) absence of neurological diseases (e.g., traumatic brain injury and epilepsy) or intracranial lesions, and (6) was not on medication for back pain within the past three months. Participants were excluded if they met one of the following exclusion criteria: (1) menstrual pain, recent/current pregnancy, or postpartum low back pain; (2) a history of spine/hip/lower limb joint surgery or injury in the past 2 years or ankle/knee instability; (3) cardio-cerebrovascular disease; (4) a history of cancer or significant unexplained weight loss; (5) suffered from depression, as defined by scoring more than 7 on the Hamilton Depression Scale, or a known psychiatric disorder that needed current pharmacotherapy; (6) cognitive deficits, as defined by scoring less than 22 on the Mini-Mental State, illiteracy, or difficulties in communication; or (7) a body mass index (BMI) of higher than 30. The healthy controls were free from symptoms of LBP for the past 6 months. The present study imposed the maximum age criteria of 65 years old due to the potential risk of falls in older adults with chronic low back pain [23] during balance test under eyes-closed condition. The interrupted visual information was detrimental to postural control in older adults [24] which increased the risk of falls in older adults to perform the balance test with eyes closed. This was also consistent with published studies which excluded adults who aged above 65 years old [25, 26]. The study also imposed the criteria to include only right-handed participants only. Published study indicated that in certain tasks, such as the sudden load task, left-handed patients showed slower response time in the nondominant side back muscles, while the right-handed patients were faster [27]. Thus, the criteria were to minimize the confounding factor of hand dominance on muscle activation in NSCLBP patients. Financial compensation was provided to all participants for the completion of the study.

### 2.2. Clinical Examination

Two licensed medical doctors confirmed the diagnosis of NSCLBP in accordance with the diagnostic guidelines published by the American College of Physicians and the American Pain Society [28]. The pain history and medical history were collected. This was followed by a physical examination and the completion of self-report questionnaires. Pain duration was measured in months.

The Chinese version of the Hamilton Depression Scale, which has good reliability with Cronbach's alpha value of 0.714 [29], was adopted to screen patients with depression. We screened out subjects with cognitive deficits by using the Chinese version of the Mini-Mental State which has shown to have good reliability with Cronbach's alpha value of 0.88 [30].

Average pain intensity in the past week was measured using the VAS (0–10 cm: “0 cm” represented no pain, while “10 cm” represented unbearable pain). The Chinese version of the Short-Form McGill Pain Questionnaire (SFMPQ) was used to measure each patient's pain experience. The main component of the SFMPQ consists of 15 items and is divided into 2 main categories: sensory scores (11 items) and affective scores (4 items) [31]. Each item was rated on an intensity scale ranging from 0 to 4 (0 = none, 1 = mild, 2 = moderate, and 3 = severe). The Chinese SFMPQ has acceptable reliability with Cronbach's alpha value of 0.664 [32].

LBP-related disability was assessed using the 100-point Chinese version of the ODI, which consists of 10 items that are each answered with a numeric value between 0 and 5 [33, 34]. The summed value was divided by the number of answered questions and then multiplied by 100%. The Chinese version of ODI has good reliability with Cronbach's alpha value of 0.78 [34].

The level of pain catastrophizing was measured by the Chinese version of the PCS. The PCS consists of 13 items, and each item was answered with a numeric value between 0 and 4; 0 corresponded to “not at all,” and 4 corresponded to “all the time” [35]. An aggregate score of pain catastrophizing was determined by summing all the item scores, with higher scores indicating a higher level of pain catastrophizing. The Chinese version of PCS and the subscales of helplessness, magnification, and rumination have good reliability with Cronbach's alpha of 0.87, 0.85, 0.62, and 0.65, respectively [35].

### 2.3. Ultrasound Measurements

Ultrasound images of bilateral TrA muscles were obtained with Sonosite M-Turbo with a linear transducer probe (6–13 MHz, B-mode, Seattle, WA, USA). To standardize the technique and to facilitate access to the abdomen, a supine crook-lying position (hips flexed to approximately 135°, knees flexed to 90°) was adopted by all of the participants [36]. The intraclass correlation coefficient (ICC) values of the measuring protocol for the percent change in thickness of TrA were 0.79–0.99 for both healthy people and people with LBP [11, 12]. While measuring the thickness of TrA at rest and during contraction, the transducer was positioned at just above the iliac crest in the midaxillary line [12]. In order to ensure measurements were recorded at the same site at rest and during contraction, we measured the thickness of TrA at rest and during contraction alternatively. Participants were instructed to slowly draw the umbilicus towards the spine and maintained the TrA contraction to enable measurement to be taken [12]. The TrA muscles were scanned 3 to 5 times bilaterally when they were at rest and contracted. Images of the TrA muscles at rest were taken at the end of exhalation; images of the contracted TrA muscles were taken during the ADIM. During the ADIM, participants were instructed to bring the umbilicus towards the spine to the greatest extent possible at the end of normal exhalation without moving the spine and to hold this position for 5 seconds with a 1-minute rest period [11, 37]. Pictures were exported for offline analysis using ImageJ (version 1.52 k, http://imagej.nih.gov/ij/) by a single examiner who was blinded to the allocation results of the participants.

**Figure 1:**
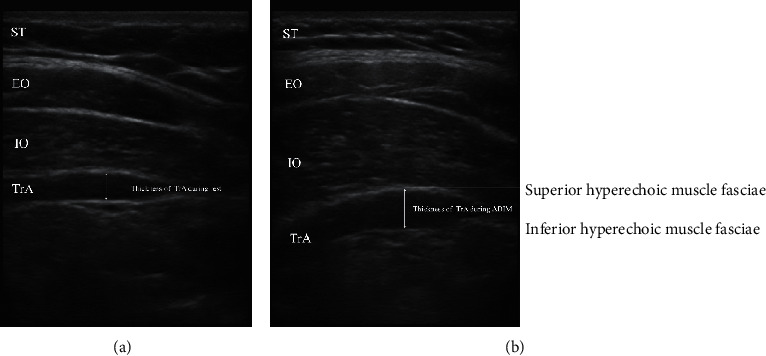
Ultrasound images and measures of transversus abdominis muscles. (a) Relaxed state. (b) Contracted state. EO: external oblique; IO: internal oblique; TrA: transversus abdominis; ST: subcutaneous tissue; and ADIM: abdominal drawing-in manoeuvre.

The perpendicular distance between the thickest parts of the superior and inferior hyperechoic muscle fasciae was measured and defined as the thickness of TrA at rest (RTrA) and during contraction (CTrA) (Figure 1). All the values of RTrA and CTrA were averaged. The percent change in thickness was calculated by using the following formula:

(1) $$\begin{array}{c} \text{\% changeTrA }=\frac{\text{CTrA}-\text{RTrA}}{\text{RTrA}}\times 100\%. \end{array}$$

### 2.4. Postural Balance Control Assessments

The COP sway area and path length were recorded by PROKIN Systems (50 Hz, PK252P, TecnoBody, Italy). A recent systematic review [7] indicated that sway area and path length were the most reliable measures of COP during balance tests in both eyes-closed and eyes-open conditions. Hence, the present study adopted COP area and COP length for the assessment of postural balance. As recommended by the manufacturer, the participants stood barefoot on a specific spot of a firm surface with their feet at an approximately 30-degree angle to the midline and 4 cm apart at the heels. All of the participants underwent 2 tests: double-leg stance with eyes-open and eyes-closed conditions. Under the eyes-open condition, the participants were instructed to stand as still as possible with their gaze fixated on a clock at a distance of 1 m. Two trials were conducted twice for every test, and each trial lasted for 30 seconds; there was a 30-second rest between each trial to prevent fatigue. The trajectory of the COP in the first 5 seconds of each trial was not recorded.

### 2.5. Reliability Measures for Balance Tests

Twenty participants from the healthy control group underwent the reliability measures for the aforementioned balance tests over 2 sessions with a 2-week interval between sessions. As all of the balance tests in this study were conducted by the same investigator; therefore, only the intrarater reliability was measured.

### 2.6. Statistical Analysis

Statistical analysis was performed with SPSS, version 26.0 (SPSS Inc., Chicago, IL, USA). The continuous variables are presented as the means ± standard deviations (SDs), and the categorical data are presented as absolute numbers. The continuous variables in healthy control group and NSCLBP group were assessed for normality by the Shapiro–Wilk test and Kolmogorov–Smirnov test, respectively. The homogeneity of the dataset was assessed by the Levene test. Log transformation was conducted to normalize the distributions of variables in the balance tests. For between-group analyses, independent-samples *t*-tests were conducted for continuous variables that were normally distributed, Mann–Whitney *U* tests were conducted for continuous variables that were not normally distributed even after log transformation, and chi-square tests were performed for categorical variables. The balance tests were analysed by repeated-measures ANOVA with 2 conditions (eyes-open and eyes-closed conditions). Independent-samples *t*-tests were conducted for those tests in which significant group interaction effects were noted. Multiple linear regression (MLR) analysis was performed to assess the multivariate relationships between static postural control parameters, clinical measures, and TrA activation in participants with NSCLBP. Only the COP variables that had significant between-group differences were established as criterion variables. MLR analysis does not allow the inclusion of variables that are associated with each other. However, each subscale of the PCS was associated with each other and the total scores of PCS. The sensory and affective scores of SFMPQ were also associated with the total scores of SFMPQ. Therefore, in the first model, we included the total scores of PCS and SFMPQ as covariates of interest in the MLR analysis. In the second model, each subscale of PCS was entered separately, while the sensory and affective scores of SFMPQ were also included as covariates of interest in the MLR analysis. The demographic variables (age, sex, weight, and height) were also included as candidate predictor variables within the MLR analyses [38]. The most parsimonious and statistically significant model was obtained using the stepwise selection method. The intrarater reliability was quantified using the intraclass correlation coefficients (ICC [3, 1]), which includes two-way random and absolute agreement. The reliability can be classified as poor (ICC < 0.4), moderate (ICC = 0.4–0.59), good (ICC = 0.6–0.75), and excellent (ICC > 0.75) [39]. The statistical significance level was set to be 0.05 for all tests.

## 3. Results

### 3.1. Demographics

During the recruitment process, 160 patients were screened for eligibility. A total of 92 patients were excluded due to the following reasons: 11 patients were not diagnosed with NSCLBP; 33 patients did not meet the criteria for duration of symptoms or severity of pain; 5 patients were excluded for history of surgery; 6 patients were excluded for cardio-cerebrovascular disease; 2 patients were excluded for history of cancer; 2 patients were excluded for cognitive deficits and difficulties in communication; 1 patient was excluded for BMI above 30; 22 patients were unable to participate because of location and time conflicts; and 10 patients declined to participate. The study included 68 participants with NSCLBP and 40 age- and sex-matched healthy controls.

The two groups did not differ significantly regarding age, gender, body mass index, weight, height, and education length (Table 1).

### 3.2. Reliability Measures

For COP sway length, the ICCs [3, 1] were 0.740 for the eyes-open condition and 0.712 for the eyes-closed condition. For COP sway area, the ICCs [3, 1] were 0.748 for the eyes-open condition and 0.728 for the eyes-closed condition. All tests showed good intrarater reliability.

### 3.3. Differences in COP Oscillation between Groups

For COP sway area, repeated-measures ANOVA revealed significant condition by group interaction effects (*F* = 9.022, *p*=0.003). The COP sway area was larger in the NSCLBP group than in the healthy control group under the eyes-closed condition (*p* < 0.001) but not under the eyes-open condition (*p*=0.270) (Table 2).

For COP sway length, repeated-measures ANOVA revealed significant condition by group interaction effects (*F* = 4.340, *p*=0.040), but no significant differences between groups were observed in any of the tests of COP sway length (*p* > 0.05) (Table 2).

### 3.4. Differences in Percent Change in TrA Thickness between Groups

The percent change in TrA thickness during the ADIM was lower in the NSCLBP group than in the healthy control group (*p* < 0.001) (Table 3 and Figure 1).

### 3.5. Multiple Linear Regression Analysis

Only the COP sway area under the eyes-closed condition showed a significant between-group difference. Hence, only the COP sway area under the eyes-closed condition was established as a criterion variable. In the first model, PCS scores, the percent change in TrA thickness on the left side, and age (*β* = 0.222, 0.341, and 0.328; *p*=0.045, 0.002, and 0.004, resp.) significantly contributed to changes in COP sway area in participants with NSCLBP (Table 4). This model explained 25.1% of the variance (*p* < 0.001)(Table 4). In the second model, the helplessness subscale of the PCS, the percent change in TrA thickness on the left side, and age (*β* = 0.236, 0.344, and 0.325; *p*=0.033, 0.002, and 0.004, resp.) significantly contributed to changes in COP sway area in participants with NSCLBP (Table 4). This model explained 25.8% of the variance (*p* < 0.001) (Table 4). The standardized positive beta coefficient indicated that larger increases in PCS scores, the percent change in TrA thickness on the left side during the ADIM, and age were associated with a larger COP sway area under the eyes-closed condition.

## 4. Discussion

To our knowledge, this study is among the first to evaluate the relationships between negative cognitive-emotional responses to pain, TrA activation, and static postural control in patients with NSCLBP. The findings of this study showed that (a) the COP sway area under the eyes-closed condition was larger in participants with NSCLBP than in healthy controls and (b) the COP area sway under the eyes-closed condition was positively correlated with PCS scores, specifically the helplessness subscale of PCS, and the activation of the left TrA in patients with NSCLBP.

### 4.1. Impaired Postural Control in NSCLBP

This study showed a larger COP sway area in the double-leg stance test under the eyes-closed condition in the NSCLBP group than in the control group. These findings are consistent with those in previous systematic reviews that reported static postural control impairment in individuals with NSCLBP under visual obstruction condition [6, 40]. One explanation may be that NSCLBP individuals have deteriorated proprioception [2, 13]. Proprioceptive information is essential for the sensorimotor system to generate motor outputs to maintain spinal posture and stability. The impairment of proprioception could engender inappropriate muscle response patterns that contribute to the disruption of balance in NSCLBP patients [2, 13, 41]. The participants relied more on the somatosensory and vestibular systems to maintain balance while standing on a stable force plate under the eyes-closed condition, whereas they may not have been able to sufficiently compensate for the loss of visual information due to impaired proprioception. Thus, when visual input was eliminated, a larger COP sway was observed in participants with NSCLBP. The findings of this study provide additional evidence that patients with NSCLBP have impaired static postural control, which may be related to an impaired proprioceptive system.

### 4.2. The Relationships between Pain Catastrophizing, Age, Transversus Abdominis Activation, and Static Postural Control

Contrary to a previous study [18] that found that pain but not fear of pain mediated alterations of postural sway in subacute LBP, we found that pain catastrophizing but not pain mediated the alteration of postural sway in patients with NSCLBP. The differences between subacute LBP and chronic LBP could contribute to this disparity. The brain activity of patients with acute/subacute LBP is primarily confined to pain-related areas, whereas chronic LBP shows a shift of brain activity from the acute pain circuitry to emotion-related circuitry [19]. Essentially, psychological factors, such as pain catastrophizing, may play a more fundamental role in chronic LBP than in subacute LBP, which would explain the observation that pain catastrophizing mediated alterations of postural control in patients with NSCLBP but not those with subacute LBP. Ruhe et al. [42] reported a significant association between pain intensity and COP parameters in chronic LBP patients but did not evaluate pain catastrophizing. Because of the different COP parameters applied, our study has limited comparability with Ruhe's study. In accordance with a study that investigated the altered neuromotor control during walking [43], our findings indicated that pain catastrophizing was more strongly associated with altered static postural control in NSCLBP patients than pain intensity.

Compared with subacute LBP patients, chronic LBP patients demonstrated higher pain intensity [44]. The increased pain intensity in chronic LBP patients is associated with increased pain catastrophizing [17], indicating that pain catastrophizing may amplify the pain intensity in chronic LBP. It was suggested that pain catastrophizing might maintain and/or intensify chronic LBP through its effects on pain-induced tension increases in trunk muscles [45]. This would in turn affect the postural control in patients with chronic LBP. Pain catastrophizing is characterized by enhanced pain perception, increased attention to noxious stimuli, the reinforcement of emotional responses to pain, or the weakened modulation of pain [46, 47]. People with greater pain catastrophizing demonstrated increased activity in cortical areas that are related to the anticipation of pain (medial frontal cortex, cerebellum), attention to pain (dorsal anterior cingulate cortex, dorsolateral prefrontal cortex), and emotional aspects of pain (claustrum) [47]. As a maladaptive strategy for coping with pain, pain catastrophizing is assumed to induce increases in muscle activity or co-contractions of trunk muscles to reduce excessive trunk motion, due to a tendency to overestimate the threat or seriousness of pain sensations [5, 14], the disengagement of cognitive resources from painful stimuli [48], and the enhancement of affective processing [14, 17]. A previous study found that the electrical activity of trunk muscles and postural sway during static standing were increased in chronic LBP patients [49]. Furthermore, pain catastrophizing was positively related to increased lumbar muscle activity, as estimated by electromyography [15], and negatively correlated with a reduced lumbar flexion range of motion [50] in patients with chronic LBP. In this study, pain catastrophizing and TrA activation were significantly correlated with COP sway area in the static balance test. These relationships were consistent with the “tight” control phenotype within NSCLBP patients. “Tight” control refers to increases in muscle contractions due to high muscle excitability that are generated to minimize excessive spinal movement [2, 5]. The findings of this study further supported the theory that pain catastrophizing may affect spinal postural control by causing muscular hyperactivity as a “guarding strategy” [5, 43]. Hence, the diversity of the postural control changes in NSCLBP patients may be partly explained by pain catastrophizing that reflects a pattern of negative cognitive-emotional responses to pain [14], which should therefore be taken into consideration when interpreting balance test results in NSCLBP patients. Furthermore, we found a negative association between the helplessness subscale of PCS and COP sway area in the static balance test. Similarly, a previous study found that the helplessness subscale of the PCS was negatively associated with the result of dynamic balance test in people with greater trochanteric pain syndrome [51]. Although only a few studies focused on the subscale of PCS and the results were inconclusive, evidence from our study was in favor of a negative association between pain helplessness and postural control. In agreement with the previous studies [52, 53], we observed age-related deterioration of postural balance, which should also be considered in the interpretation of balance tests.

Deep core muscles, such as the transversus abdominis (TrA), are part of the active zone and play an essential role in postural stability [9]. Ultrasound imaging studies have indicated that there is a moderate correlation between postural balance and the thickness of the right TrA during rest and the ADIM in participants with chronic LBP [10]. However, recent systematic reviews [54, 55] have indicated that body mass index (BMI), age, sex, height, and posture are all confounders of the thickness of deep core muscles during rest and contractions. It would be inappropriate to include BMI, age, sex, height, and thickness of TrA muscles during rest and contractions as predictor variables in MLR analysis. Furthermore, the ultrasound-measured percent change in muscle thickness is significantly correlated with muscle electrical activity [12] and is commonly used to compare the activity of trunk muscles between healthy controls and patients with spinal pain [56]. Hence, we only measured the percent change in muscle thickness and used it as a candidate predictor variable in the multivariate linear regression analyses.

Our study suggested that, compared with the healthy individuals, the participants with NSCLBP in this study showed reduced TrA voluntary activation during the ADIM, which is consistent with the results in a previously published study [39]. However, only TrA activation on the left side was correlated with postural control impairment in participants with NSCLBP. A study by Emami et al. reported that only decreased thickness of the right TrA during rest and contractions was correlated with poor dynamic postural balance [10]. The authors of the study did not offer specific explanations. The thicknesses of TrA muscles during rest or contractions did not differ by hand dominance [57]. Thus, the underlying mechanism for this observation is inclusive. This result is probably due to the more pronounced delay in the activation of the left TrA/internal oblique during symmetric tasks in NSCLBP patients than in controls, as a delay in the activation of trunk muscles may contribute to postural instability [58].

## 5. Clinical Implications

The results of this study showed that pain catastrophizing may affect static balance in patients with NSCLBP, implying that pain catastrophizing may influence postural control in patients with NSCLBP. It may be appropriate to combine motor control exercises with interventions that target pain catastrophizing and other psychological factors to optimize treatment outcomes for patients with NSCLBP.

## 6. Limitations

The findings of the present study should be interpreted with caution due to some limitations. First, this study investigated TrA activation during the ADIM but not during a static balance task. Thus, the results of the relationships between pain catastrophizing and TrA activation in the balance tests are limited. However, measuring TrA activation by ultrasonography during the balance trials was not technically feasible and likely affected the results. Second, the sample size of the present study was not power-calculated. Thus, it is likely to contain type II error. Further study of sufficient power should be conducted to confirm the findings of the present study. Third, this study investigated the activation of the TrA but not the multifidus muscles, which also play an essential role in postural control. Additional studies that include measurements of multifidus may allow us to gain a deeper understanding about how the activation of multifidus influences static postural control in people with NSCLBP. Last but not least, because of the cross-sectional design of this study, more cohort studies are needed to further investigate the relationships between postural control and pain catastrophizing and core muscle activation in the future.

## 7. Conclusion

The results of this study showed that impaired static postural control was evident under the visual deprivation condition and the static postural control in NSCLBP patients was correlated with pain catastrophizing and voluntary TrA activation. These relationships suggest that pain catastrophizing may also affect static postural control in patients with NSCLBP and should be taken into consideration in the interpretation of balance test results and the management of NSCLBP. Additional studies should be conducted to investigate the underlying biological and psychosocial interactions to explore the multidimensional nature of NSCLBP.

## Figures and Tables

**Table 1:** Temperature and wildlife count in the three areas covered by the study.

Characteristics	HC (*n* = 40) (range)	NSCLBP (*n* = 68) (range)	*p* value
Male/female (*n*)†	14/26	21/47	0.659
Age (years)	28.725 ± 7.633 (20–54)	30.102 ± 8.974 (20–56)	0.439
BMI (Kg/m2)	20.696 ± 2.344 (16.61–24.98)	21.678 ± 3.035 (17.26–33.50)	0.084
Weight (Kg)	57.273 ± 9.781 (40–80)	58.710 ± 9.465 (42–89)	0.407
Height (m)	1.659 ± 0.087 (1.50–1.83)	1.645 ± 0.075 (1.50–1.80)	0.377
Education length (years)	17.100 ± 1.864 (9–21)	17.735 ± 2.063 (11–23)	0.138
Pain duration (months)	N/A	38.544 ± 41.609 (3–192)	N/A
VAS (0–10 cm)	N/A	5.793 ± 1.334 (2–8.5)	N/A
ODI (0–100) (%)	N/A	14.412 ± 7.026 (0–32)	N/A
PCS (0–52)	N/A	12.544 ± 8.519 (0–34)	N/A
PCS_H (0–20)	N/A	3.353 ± 2.986 (0–14)	N/A
PCS_M (0–20)	N/A	5.279 ± 3.648 (0–13)	N/A
PCS_R (0–44)	N/A	10.427 ± 7.388 (0–31)	N/A
SFMPQ (0–45)	N/A	9.000 ± 4.333 (2–25)	N/A
SFMPQ_A (0–12)	N/A	3.585 ± 2.228 (0–9)	N/A
SFMPQ_S (0–33)	N/A	5.485 ± 3.059 (2–16)	N/A

BMI: body mass index, HC: healthy control, NSCLBP: nonspecific chronic low back pain, ODI: Oswestry Disability Index, PCS: Pain Catastrophizing Scale, PCS_H: helplessness subscale of Pain Catastrophizing Scale, PCS_M: magnification subscale of Pain Catastrophizing Scale, PCS_R: rumination subscale of Pain Catastrophizing Scale, SFMPQ: Short-Form McGill Pain Questionnaire, SFMPQ_A: affective subscale in Short-Form McGill Pain Questionnaire, SFMPQ_S: sensory subscale in Short-Form McGill Pain Questionnaire, SFMPQ: Short-Form McGill Pain Questionnaire, VAS: Visual Analogue Scale, and N/A: not applicable. Data was represented as mean ± standard deviation unless otherwise indicated. †Chi-square.

**Table 2:** COP sway variables under 2 conditions contracting the two groups.

Group	COP sway area (mm^2^)		COP sway length (mm)	
	EO condition (mean ± SD)	EC condition (mean ± SD)	EO condition (mean ± SD)	EC condition (mean ± SD)
HC (*n* = 40)	166.263 ± 8.676	242.800 ± 115.863	240.213 ± 60.605	367.125 ± 86.984
NSCLBP (*n* = 68)	191.147 ± 122.964	354.485 ± 191.661	247.029 ± 92.283	397.059 ± 174.873
*p*value	0.270	<0.001^*∗*^	0.726	0.374

COP: center of pressure, EO: eyes-open, EC: eyes-closed, NSCLBP: nonspecific chronic low back pain, and HC: healthy control. Significant *p* value is marked with^*∗*^.

**Table 3:** TrA percent thickness change contrasting the two groups.

Group	TrA percent thickness change (%)	
	Left TrA (mean ± SD)	Right TrA (mean ± SD)
HC (*n* = 40)	88.754 ± 33.823	96.455 ± 46.054
NSCLBP (*n* = 68)	45.628 ± 22.722	45.532 ± 26.679
*p* value	<0.001^*∗*^	<0.001^*∗*^

TrA: transversus abdominis; NSCLBP: nonspecific chronic low back pain, and HC: healthy control. Significant *p* values are marked with^*∗*^.

**Table 4:** Multiple linear regression for clinical measures and pain catastrophizing.

Criterion variable: COP area sway under eyes-closed condition				
Model 1				
*R* ^2^ = 0.251	Adjust *R*^2^ = 0.216		*F* = 7.147	*p* < 0.001^*∗*^
Predictor variables	Regression coefficient, B	Standardized coefficient, *β*	*p* value	VIF
PCS	4.996	0.222	0.045	1.012
lTrA%	287.755	0.341	0.002	1.003
Age	7.095	0.328	0.004	1.009

Excluded variables				
SFMPQ	N/A	−0.002	0.987	1.121
VAS	N/A	0.124	0.280	1.105
ODI	N/A	−0.158	0.207	1.325
rTrA	N/A	−0.156	0.231	1.428
Height	N/A	0.132	0.238	1.050
Weight	N/A	0.079	0.479	1.046
Gender	N/A	0.068	0.541	1.035

Model 2				
*R* ^2^ = 0.258	Adjust *R*^2^ = 0.223		*F* = 7.400	*p* < 0.001^*∗*^
Predictor variables	Regression coefficient, B	Standardized coefficient, *β*	*p* value	VIF
PCS_H	15.168	0.236	0.033	1.009
lTrA%	290.203	0.344	0.002	1.004
Age	7.016	0.325	0.004	1.006

Excluded variables				
SFMPQ_S	N/A	0.051	0.658	1.100
SFMPQ_A	N/A	−0.25	0.820	1.048
VAS	N/A	0.126	0.268	1.100
ODI	N/A	−0.138	0.256	1.259
rTrA%	N/A	−0.144	0.267	1.420
Height	N/A	0.139	0.213	1.053
Weight	N/A	0.121	0.289	1.116
Gender	N/A	0.072	0.518	1.036

COP: center of pressure, VAS: Visual Analogue Scale, ODI: Oswestry Disability Index, PCS: Pain Catastrophizing Scale, PCS_H: helplessness subscale of Pain Catastrophizing Scale, SFMPQ: Short-Form McGill Pain Questionnaire, SFMPQ_A: affective subscale in Short-Form McGill Pain Questionnaire, SFMPQ_S: sensory subscale in Short-Form McGill Pain Questionnaire, lTrA%: left transversus abdominis percent thickness change, rTrA%: right transversus abdominis percent thickness change, and N/A: not applicable. Significant *p* values are marked with^*∗*^.

## Data Availability

The data used and analysed during the current study are available from the corresponding authors upon reasonable request.
